# Spatial and space–time clustering of mortality due to malaria in rural Tanzania: evidence from Ifakara and Rufiji Health and Demographic Surveillance System sites

**DOI:** 10.1186/s12936-015-0905-y

**Published:** 2015-09-26

**Authors:** Majige Selemani, Sigilbert Mrema, Amri Shamte, Josephine Shabani, Michael J. Mahande, Karen Yeates, Amina S. Msengwa, Maurice C. Y. Mbago, Angelina M. Lutambi

**Affiliations:** Department of Statistics, University of Dar es Salaam, P.O. Box 35047, Dar es Salaam, Tanzania; Ifakara Health Institute, (IHI), Plot 463, Kiko Avenue, Off Old Bagamoyo Road, Mikocheni, P.O Box 78373, Dar es Salaam, Tanzania; Department of Epidemiology and Applied Biostatistics, Kilimanjaro Christian Medical University College, Moshi, Tanzania; Department of Medicine, Queen’s University, 94 Stuart Street, Kingston, Canada

**Keywords:** Spatial methods, Malaria mortality, Clustering

## Abstract

**Background:**

Although, malaria control interventions are widely implemented to eliminate malaria disease, malaria is still a public health problem in Tanzania. Understanding the risk factors, spatial and space–time clustering for malaria deaths is essential for targeting malaria interventions and effective control measures. In this study, spatial methods were used to identify local malaria mortality clustering using verbal autopsy data.

**Methods:**

The analysis used longitudinal data collected in Rufiji and Ifakara Health Demographic Surveillance System (HDSS) sites for the period 1999–2011 and 2002–2012, respectively. Two models were used. The first was a non-spatial model where logistic regression was used to determine a household’s characteristic or an individual’s risk of malaria deaths. The second was a spatial Poisson model applied to estimate spatial clustering of malaria mortality using SaTScan™, with age as a covariate. ArcGIS Geographical Information System software was used to map the estimates obtained to show clustering and the variations related to malaria mortality.

**Results:**

A total of 11,462 deaths in 33 villages and 9328 deaths in 25 villages in Rufiji and Ifakara HDSS, respectively were recorded. Overall, 2699 (24 %) of the malaria deaths in Rufiji and 1596 (17.1 %) in Ifakara were recorded during the study period. Children under five had higher odds of dying from malaria compared with their elderly counterparts aged five and above for Rufiji (AOR = 2.05, 95 % CI = 1.87–2.25), and Ifakara (AOR = 2.33, 95 % CI = 2.05–2.66), respectively. In addition, ownership of mosquito net had a protective effect against dying with malaria in both HDSS sites. Moreover, villages with consistently significant malaria mortality clusters were detected in both HDSS sites during the study period.

**Conclusions:**

Clustering of malaria mortality indicates heterogeneity in risk. Improving targeted malaria control and treatment interventions to high risk clusters may lead to the reduction of malaria deaths at the household and probably at country level. Furthermore, ownership of mosquito nets and age appeared to be important predictors for malaria deaths.

**Electronic supplementary material:**

The online version of this article (doi:10.1186/s12936-015-0905-y) contains supplementary material, which is available to authorized users.

## Background

Knowledge about when and where malaria mortality clustering occurs in the populations is essential for targeting malaria interventions and resource allocation. Malaria morbidity and mortality in Sub-Saharan African (SSA) region has been declining [1]. A recent Tanzanian 2011–2012 HIV/AIDS and Malaria Indicator Survey report showed a decline in under-five malaria prevalence in Tanzania Mainland [2]. Reduction in malaria prevalence and deaths is an essential step towards increased efforts to accelerate progress towards achievement of millennium development goals (MDG) 4 and MDG 6. However, achieving these goals requires better understanding on geographical malaria distribution and factors that influence high-risk for malaria deaths.

Several studies have mapped the distribution of high risk area for malaria and have identified populations at risk at continental/country level [3–6]. These studies and several others [5–7] have used data on prevalence of infection collated by the Mapping Malaria Risk in Africa project (MARA), while others have used hospital data to explore the burden of malaria [8, 9]. However, fewer studies have investigated on the risk factors of malaria mortality using verbal autopsy data [10–12]. The results of these studies suggested that age, community awareness for early treatment and scale up use of mosquito net are predictors of malaria mortality. Finding from a randomised controlled trial revealed that mosquito net does not reduce malaria transmission and mortality in high transmission area [13]. Recognizing malaria clustering, hotspot and coldspot for malaria deaths would permit control efforts to be directed to specific geographic areas, reducing costs and increasing effectiveness [14]. Control of malaria in such hotspots might also eventually lead to elimination of deaths related to malaria.

Measuring malaria burden in a community is a challenge to most developing countries including Tanzania [15, 16], because most disease incidence and deaths occur outside the formal health care system [17, 18], where no records are available. As a result, verbal autopsy (VA) is currently an alternative approach to determine malaria-specific death [19, 20]. VA is a method used to ascertain the cause of a death based on interview with next of kin or other caregivers. This is done using a standardized questionnaire that obtains information on signs, symptoms, medical history and circumstances preceding death [21]. VA procedures have been evaluated in SSA and other countries [22–24]. Results from these studies concluded that VA is a reliable estimate for specific cause of deaths [24]. Moreover, other studies have evaluated the validity of VA on determining malaria specific mortality [24, 25] and concluded that VA methods have an acceptable level of diagnostic accuracy at the community level.

In this study, VA data generated from Rufiji and Ifakara HDSS in rural Tanzania were used. These HDSS sites are among sites established within the INDEPTH network of many Sub-Saharan African countries which provide evidence-based health information on monitoring the impact on various policies in the population [26]. Although, the two HDSS sites are small to represent the whole country, no attempt documented malaria mortality patterns and trend in Tanzania for at least 10 years period. Also, use of spatial techniques for identifying clustering for malaria specific cause of death in Tanzania is unclear. Investigating spatial and space–time clustering of malaria mortality, would provide evidence for evaluation of the impact on malaria control interventions in achievement of millennium development goals (MDG) 4 for child survival and MDG 6 (for combating HIV/AIDS, malaria and other diseases).

This study aimed at examining spatial and spatio-temporal trends and clustering of mortality due to malaria in Rufiji HDSS (1999–2011) and Ifakara HDSS (2002–2012). In addition, the study aimed at identifying individual, households and other factors related to malaria mortality risks and map their spatial clustering relative to malaria mortality hotspots and coldspots for appropriate planning and control measures against malaria.

## Methods

### Study area

The study was carried out in Rufiji and Ifakara Health and Demographic Surveillance System (HDSS) sites. Both sites are located in the Greater Rufiji River Basin in southern Tanzania [27]. The sites are primarily rural with majority of the population relying on subsistence farming or fishing. Both sites are characterized by heavy rains from March to May. These two HDSS sites were selected because they are among the HDSS sites which continuously collect large amounts of data on defined geographical areas and longitudinal data for malaria specific cause of death in Tanzania. Based on microscopy testing for the health facility survey in 2012 [28], these HDSS sites still have high malaria prevalence of approximately 19.2 % in high season and 7.2 % in low season in Rufiji HDSS and 9.4 % in high season and 4.2 % in low season in Ifakara HDSS.

### Rufiji HDSS site

**Fig. 1:**
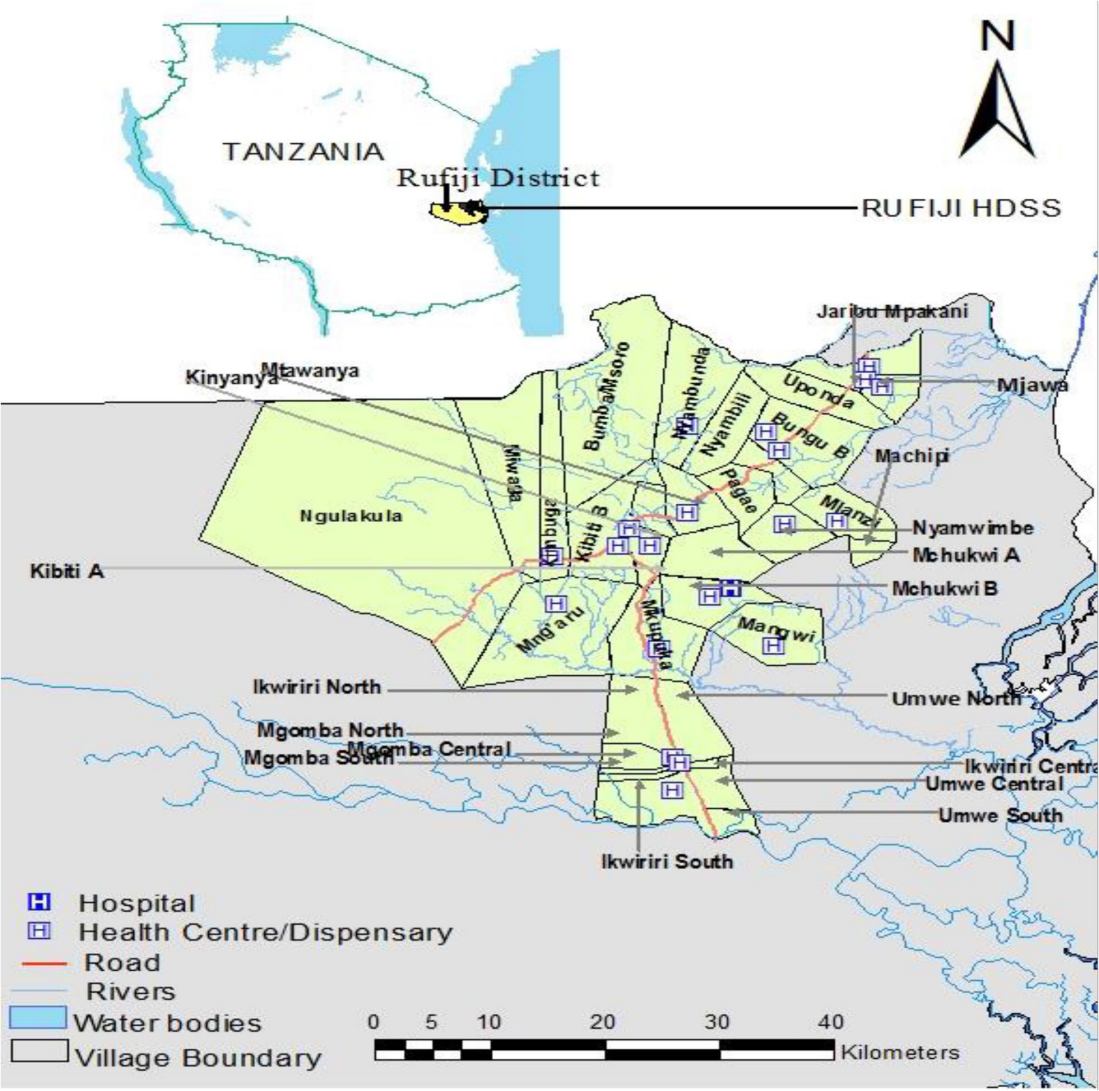
Reference map for Rufiji Health and Demographic Surveillance System site

Rufiji HDSS is situated in Rufiji District, Coast region, with 38 villages covering an area of 1814 km^2^. The Rufiji HDSS is located in eastern Tanzania 7.45°–8.03° south latitude and 38.62°–39.17° east longitude (Fig. 1). The vegetation of the HDSS is formed mainly by tropical forests and grassland. The weather is hot throughout the year and with rainy seasons. The average annual precipitation in the district is between 800 and 1000 mm. The population size of the Rufiji HDSS is approximately 103,503 people living in 19,315 households [29]. The HDSS is largely rural and highly populated in centres along the main roads. The Rufiji HDSS has 24 health facilities in the surveillance area (one non-government hospital, two health centres and 21 dispensaries).

### Ifakara HDSS site

**Fig. 2:**
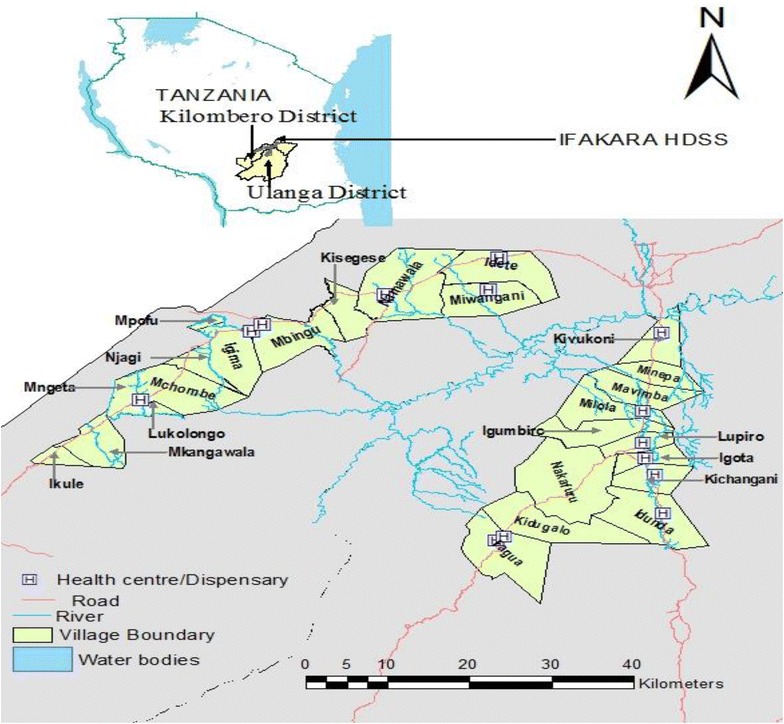
Reference map for Ifakara Health and Demographic Surveillance System site

Ifakara HDSS is situated in and covers parts of Kilombero and Ulanga Districts in Morogoro Region. The Ifakara HDSS covers 25 villages (13 in Kilombero and 12 in Ulanga districts) in Morogoro region south-eastern part of the country. The Ifakara HDSS is located in eastern Tanzania 8.0°–8.58° south latitude and 36.00°–36.80° east longitude (Fig. 2). The HDSS site constitutes more than 124,000 people, living in 28,000 scattered rural households [30]. The two districts are divided by the extensive floodplains of the Kilombero River, a potentially high risk and malaria endemic area (Fig. 2). The HDSS is predominantly rural with an ethnically heterogeneous population that practice subsistence farming, fishing and small scale trading. The population of the Ifakara HDSS area is served by a network of health facilities and at the time of the study, there were 14 health facilities (two health centres and 12 dispensaries).

### Study design and data collection

The analysis used data collected in Rufiji and Ifakara HDSS sites. Individual and yearly malaria deaths were extracted from the Rufiji and Ifakara HDSS database covering a period of January 1999 to December, 2011 and January 2002 to December 2012, respectively. The two HDSS sites have consistently been recording pregnancies, pregnancy outcomes, deaths and migrations by visiting households once every 4 months since 1997 in Ifakara HDSS and 1998 in Rufiji HDSS. Household registers are used to record each of those events. All registered deaths are followed up with a verbal autopsy (VA) form by well trained field staff. Date of birth of each individual is included in the household registers and each event is recorded along with the specific date it occurred.

Data credibility in HDSS was ensured at all stages of collection and processing to enhance quality. Up to 5 % of randomly selected households were visited by field supervisors for repeated interviews. Other strategies including accompanied interviews as well as surprise field visits by field supervisor. Data management was done using the household-registration system (HRS 2) with in-built consistency and range checks.

### Verbal autopsy procedure

The WHO and INDEPTH Network [31] standardized VA questionnaire was adapted and used for data collection on causes of death. In the HDSS, deaths were captured during rounds of update. Then HDSS field interviewers visited the deceased’s home after a grieving period to administer a verbal autopsy questionnaire. An interview was administered to relatives or caregivers who were closely associated with the deceased during the period leading to his or her death. The questionnaire assessed the identity of the deceased and established the sequence of events leading to death, including symptoms and signs of the illness before death. Verbal autopsy was carried out since 1998 in Rufiji HDSS and 2002 in Ifakara HDSS. The verbal autopsy forms are independently reviewed by two physicians according to a list of causes of death based on the 10th revision of the International Classification of Diseases (ICD-10). A third physician is asked to code the cause of death in the case of discordant results. If there is disagreement among the three physicians, the death is coded as “undetermined” cause [32]. Causes of death (main, immediate, and/or contributing) are coded to be consistent with the ICD-10 [33]. Malaria deaths are coded as direct when malaria is the underlying cause of death or indirect when malaria is one of several diseases leading to death but the death is attributed by a different cause) [34].

### Geo-referencing location of households and health facilities

The geographic information available in the HDSS database included the coordinates (longitude and latitude) and altitude of the majority of the households, health facilities and village. These were collected on-site using handheld global positioning system (GPS) receivers or tablet with in-built GPS reader at a precision of less than 10 m [35]. Twenty percent of households were not geo-located in HDSS database and were primarily collected using handheld global positioning system and mapped in a geographic information system (GIS) database.

### Data processing and analysis

All-cause mortality data were obtained from the Rufiji and Ifakara HDSS database for the period 1999–2011 and 2002–2012, respectively. Individual-specific information extracted from the HDSS database includes date of birth, start and exit from the study, age, sex, mosquito net ownership, socio-economic status and death status. Other information such as location of the household, health facility and altitude were obtained in HDSS database and were collected by other projects that were carried out within the HDSS platforms, and few households with missing coordinates were primarily geo-located. Nearest distance to health facility was calculated using the spherical law of cosines for straight line using the latitudes and longitudes of health facilities and households. The formula (as described in detail in [36]) was used. Nearest distance to health facility was classified into two groups: less than 5 km and 5 km and above [37].

Person time at risk (person-years) contributed by each person was calculated until exit. Exit from the study was due to migration (outside the HDSS area), death or end of the study. In a case where a person migrated to a different household location within the study area, time at risk was computed separately for new location and added to the total time at risk. The outcome of interest is the death status of an individual or total monthly/yearly death for specific age groups (age group was categorized into under five and five and above). The malaria mortality rates were calculated by dividing the number of deaths by the person-years of observation and were expressed per 1000 person-years (py). Seasons at death were classified into two groups: dry (June–October) and wet (November–May) according to the dates of dying that correspond to the seasons of the year in the study area.

Household wealth status was constructed using the principal component analysis (PCA) method [38]. Items included in the PCA were household assets such as animals; TV, bicycle and radio and household characteristics such as the type of toilet, source of drinking water, house roofing material, wall material, and floor material were included. Finally, all households were classified into five categories: poorest, poorer, poor, less poor or least poor, according to their household wealth score. The outcome variable, death due to malaria, was defined by assigning “1” if a person died due to malaria or “0” if a person had not died due to malaria. The explanatory variables were age, distance to nearest health facility, sex, season, social economic status (SES), ownership of mosquito nets, and altitude.

### Malaria control interventions

**Fig. 3:**
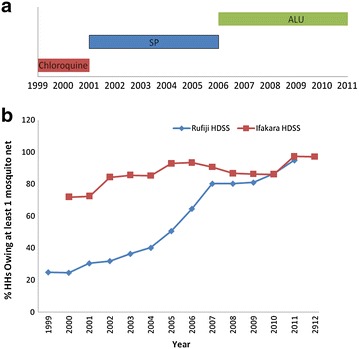
Major malaria control interventions in the study areas. **a** First line malaria treatment policy, **b** proportion of households with at least one mosquito nets in Rufiji and Ifakara HDSS

The HDSS sites routinely collect data on mosquito net ownership (household ownership mosquito nets). Policy for malaria treatment changes were extracted from existing publications. In August 2001, the first-line malaria treatment policy changed from chloroquine to sulphadoxine–pyrimethamine (SP) [39]. Artemisinin-based combination therapy (ACT) was introduced in January 2007 through a change from SP [40]. The start date of the IMCI interventions was set in April 2002 in Kilombero and Ulanga, and 1997 in Rufiji District. Figure 3 shows the coverage of these malaria control interventions over time.

### Statistical method

#### Modelling the relationship between malaria mortality and risk factors

Statistical analysis and model building were performed using STATA software (version 11, College Station, TX, USA), using survey procedures that account for clustering and stratification. The analysis used both descriptive and analytical statistics. Dying due to malaria rates by each variable were calculated and presented. Pearson’s Chi Square test was used to determine the association between a set of explanatory variables and dying due to malaria for categorical variables. Further analyses for all variables were individually analyzed using logistic regression with villages as random effects to account for clustering. All percentages and odds ratios reported are population-average estimates which have been adjusted to take into account the clustering at village level. Selection of variables for inclusion in the multivariate model was based on the log-likelihood ratio test, whereby a variable was retained in the model if there was statistical evidence that its presence improved the model and possible association with dying by malaria (p < 0.2) in the univariate analysis model [41]. The model was finally checked for presence of interaction and adequacy before being approved as final.

#### Clustering for mortality due to malaria

SaTScan™ software version 9.3, using Martin Kulldorff method [42], was used to identify the geographical clusters with high mortality due malaria using Poisson model. The package has been used by researchers [43, 44] to determine the frequency or rate of occurrence and the extent to which such events occurred over a specified period of time within a defined area and population. The analysis was purely spatial, purely temporal or space–time. This methodology identifies clusters with higher numbers of observed cases (malaria deaths) than expected cases under spatial randomness, and then evaluates their statistical significance by gradually scanning a circular window that spans the area of study. A likelihood ratio test compares the observed deaths of the disease within the circle to the expected deaths across the entire range to identify significant clusters of disease, providing relative risk and p values for any clusters identified [42]. The model was run with a maximum cluster size of 50 % of the total population and p values generated across 999 Monte Carlo replications to ensure no loss of power at the alpha = 0.05 level [42].

Two local measures of spatial association were used within ArcGIS 10.1 to indicate “where the clusters or outliers are located” and “what type of cluster and intensity is most important” [45]. Anselin Local Moran’s I [45] was used to detect core clusters/outliers of villages with extreme malaria mortality rate values unexplained by random variation, and to classify them into hotspots (high values next to high, HH), cold spots (low values next to low, LL) and spatial outliers (high amongst low, HL or vice versa, LH). Local Moran’s I tests the null hypothesis of absence of spatial clustering of malaria mortality in the villages of the study areas (for polygon features) when its expected value is −1/(N − 1). This method has been used in other studies to identify HIV prevalence hotspots [46, 47], and malaria hotspots in particular [14].

Further, the local Getis-Ord statistic (Gi*) was used to provide additional information indicating the intensity and stability of core hotspot/cold spot clusters [47, 48] for significant predictors variables. Gi* statistic identifies different spatial clustering patterns like hotspots, high risk and cold spots over the entire study area with statistical significance [48]. The statistic returns a Z score for each feature in the dataset. For statistically significant positive Z score, the larger the Z score is, the more intense the clustering of high values (hot spots). For statistically significant negative Z score, the smaller the Z score is, the more intense the clustering of low values (cold spots). High risk areas are at lower significance level in comparison to hot spots. Villages with Z scores >2.58 were considered significant at 99 % confidence level (P < 0.01) and were put in the hot spot category. Villages with Z scores between 1.65–1.96 and 1.96–2.58 were considered significant at 90 and 95 % confidence level (P < 0.10 and 0.05) and were categorized as high risk villages. Z scores <−2.58 indicated clustering of low values and were considered as cold spots [14]. The Getis-Ord Gi* index was calculated as described in [47]. Results were mapped using geographical information system (GIS) (ArcGIS, version 10.1, CA, USA). The Tanzania administrative boundaries were downloaded from National Bureau of Statistics website and added to the map as a layer.

### Ethical approval

The Ethical clearance was granted by the Ifakara Health Institute (IHI)’s Institutional Review Board (IRB), Tanzania and Medical Research Coordinating Committee (MRCC) of the National Institute for Medical Research (NIMR) for the establishment of Rufiji and Ifakara HDSS. For each household visit, verbal consent was sought from the respondent.

## Results

### Descriptive results

The analysis included 11,462 deaths that occurred in Rufiji HDSS for the period 1999–2011. Of these, more than half (51 %) were female and over two-thirds (69 %) of deaths were 5 and above years old in Rufiji HDSS. Seasonal variations in deaths were observed whereby more deaths occurred during wet season (Table 1).

**Table 1:** Background characteristics for all causes of death in the study areas

Variable	Rufiji HDSS (N = 11462)		Kilombero/Ulanga (N = 9328)	
	All causes (%)	95 % CI	All causes (%)	95 % CI
Sex				
Male	5613 (49.0)	47.6–50.3	4750 (50.9)	50.0–51.9
Female	5849 (51.0)	49.7–52.4	4578 (49.1)	48.1–50.0
Age				
Under 5	3505 (30.6)	28.0–33.3	3583 (38.4)	34.9–42.0
5 and above	7957 (69.4)	66.7–72.0	5745 (61.6)	58.0–65.1
Ownership of ITN				
Yes	5198 (45.4)	42.5–48.2	7157 (76.7)	74.6–78.7
No	6264 (54.6)	51.8–57.5	2170 (23.3)	21.3–25.4
Distance to nearest HF				
Less than 5 km	3366 (30.1)	21.3–40.6	4996 (55.6)	43.5–67.1
5 and more than 5 km	7828 (69.9)	59.4–78.7	3996 (44.4)	32.9–56.6
Social economic status				
Poorest	2404 (21.0)	18.9–23.3	2253 (24.2)	22.0–26.4
Poorer	2433 (21.2)	19.5–23.1	1772 (19.0)	17.8–20.3
Poor	2301 (20.1)	19.1–21.1	2114 (22.7)	21.8–23.5
Less poor	2342 (20.4)	19.1–21.9	1746 (18.7)	17.5–20.0
Least poor	1982 (17.3)	14.8–20.2	1443 (15.5)	13.6–17.6
Season				
Wet season	7572 (66.1)	65.1–67.0	5436 (58.3)	57.0–59.6
Dry season	3890 (33.9)	33.0–34.9	3892 (41.7)	40.4–43.0

As shown in Table 2, malaria deaths were 23.6 % in Rufiji HDSS. Overall, deaths among under-five children accounted for 30.6 % of all causes of death of whom 33.1 % (95 % CI: 31.2–34.9) were malaria related deaths in Rufiji. The proportional death attributable to malaria was 27.9 % in households without mosquito net at death in Rufiji. A greater proportion of under-fives died as a result of malaria compared to those aged five and above, this difference was statistically significant in Rufiji and Ifakara HDSS (Table 2).

**Table 2:** Distribution of malaria deaths by explanatory variables in the study areas

Variable	Rufiji HDSS		P value	Kilombero/Ulanga		P value
	Malaria death n/N (%)	95 % CI		Malaria death n/N (%)	95 % CI	
Sex	2699/11,462 (23.6)	22.8–24.3	<0.01	1596/9328 (17.1)	16.4–17.9	0.409
Male	1277/5613 (22.8)	21.5–24.1		795/4750 (16.7)	15.0–18.7	
Female	1422/5849 (24.3)	22.9–25.8		801/4578 (17.5)	16.1–19.0	
Age	2699/11,462 (23.6)	22.8–24.3	<0.01	1596/9328 (17.1)	16.4–17.9	<0.01
Under 5	1159/3505 (33.1)	31.2–34.9		896/3583 (25.0)	23.4–26.7	
5 and above	1540/7957 (19.4)	18.3–20.5		700/5745 (12.2)	11.2–13.2	
Ownership of ITN	2699/11,462 (23.6)	22.8–24.3	<0.01	1596/9328 (17.1)	16.4–17.9	<0.01
Yes	953/5198 (18.3)	16.9–19.9		1148/7157 (16.0)	14.8–17.5	
No	1746/6264 (27.9)	26.5–29.3		448/2170 (20.6)	18.3–23.2	
Distance to nearest HF	2548/11,194 (22.8)	21.6–24.0	0.156	1558/8992 (17.3)	15.9–18.9	0.330
Less than 5 km	712/3366 (21.2)	18.6–24.0		836/4996 (16.7)	15.0–18.7	
5 and more than 5 km	1836/7828 (23.5)	22.2–24.8		722/3996 (18.1)	15.9–20.4	
Social economic status	2699/11,462 (23.6)	22.8–24.3	0.177	1596/9328 (17.1)	16.4–17.9	0.554
Poorest	557/2404 (23.2)	21.3–25.2		362/2253 (16.1)	14.2–18.1	
Poorer	612/2433 (25.2)	23.5–26.9		336/1771 (19.0)	16.6–21.7	
Poor	536/2301 (23.3)	21.3–25.4		363/2115 (17.2)	15.4–19.1	
Less poor	556/2342 (23.7)	21.8–25.8		303/1746 (17.4)	15.6–19.3	
Least poor	438/1982 (22.1)	20.2–24.1		232/1443 (16.1)	14.3–18.0	
Season	2699/11,462 (23.6)	22.8–24.3	0.035	1596/9328 (17.1)	16.4–17.9	0.043
Wet season	1826/7572 (24.1	22.8–25.5		971/5436 (17.9)	16.3–19.6	
Dry season	873/3890 (23.6)	21.1–23.9		625/3892 (16.1)	14.5–17.7	

In Ifakara HDSS, a total of 9328 deaths occurred and 49.1 % were female. More than 60 % in Ifakara were aged 5 and above years (Table 1). The malaria related deaths contributed to 17.1 % for all deaths that occurred in Ifakara HDSS during the study period. Overall, deaths among under-five children accounted for 38.4 % of all causes of death of whom 25 % (95 % CI: 23.4–26.7) was malaria related deaths in Ifakara HDSS. The distribution of malaria deaths by sex, season, socio-economic statuses, ownership of mosquito net, distance of household to the nearest health facility are shown in Table 2. In terms of socio-economic status, no evidence of inequality between quintiles and malaria deaths was found in both sites.

### Spatial clusters of malaria mortality

**Fig. 4:**
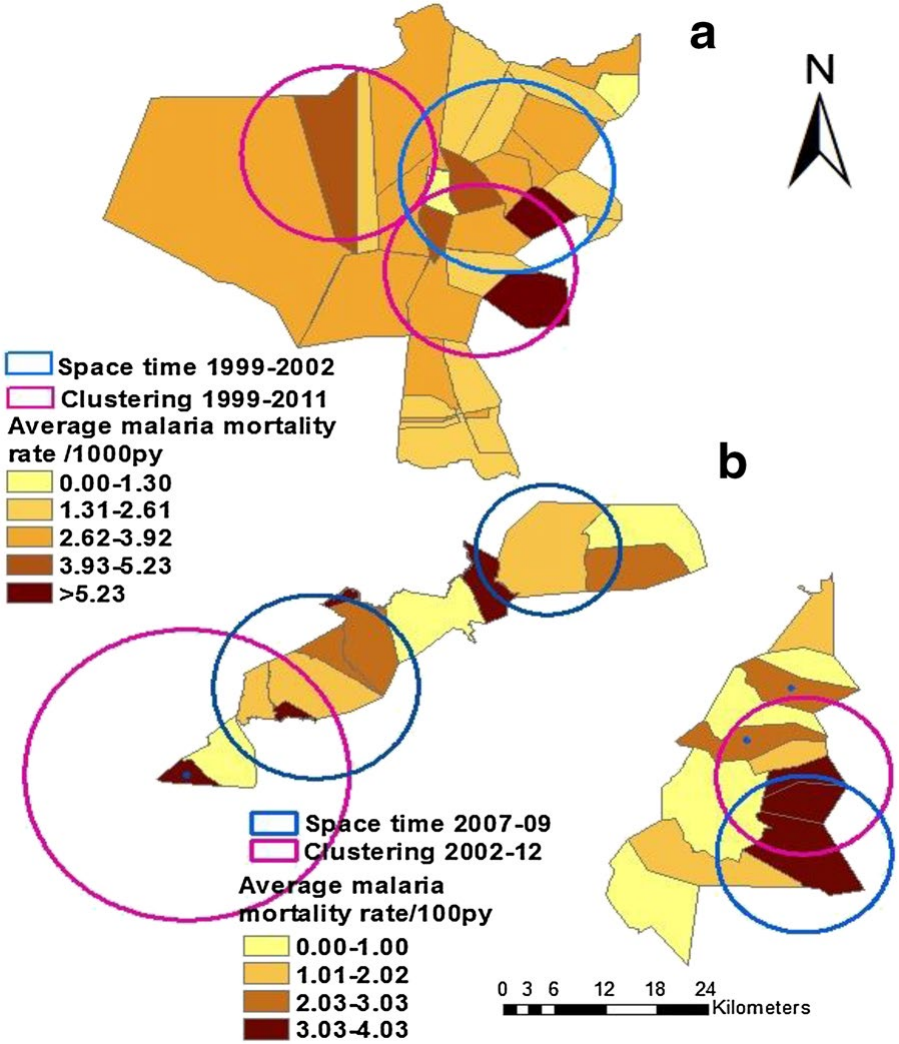
Geographical clustering and space time clustering for estimated malaria mortality rate in Rufiji and Ifakara HDSS by village. **a** For Rufiji HDSS and **b** for Ifakara HDSS

Spatial scan statistic indicated village’s spatial clusters of malaria death for 1999–2011 in Rufiji HDSS (Fig. 4). The purely spatial analysis (Table 3) revealed three significant clusters, the first cluster was in 2005 and involved 10 villages (Mtawanya, Kinyanya, Pagae, Mchukwi A, Kibiti A, Kibiti B, Nyambili, Nyamwimbe, Mchukwi B, Bungu B) with 112 total malaria deaths cases and 81 expected cases under the null hypothesis of no clusters (RR = 2.10, p < 0.001); the second significant cluster was observed in 2006 and consisted of 11 villages that include Pagae, Bungu A, Mtawanya, Nyambili, Mchukwi A, Bungu B, Kinyanya, Nyamwimbe, Mlanzi, Kibiti A, Nyambunda (RR = 1.71, p < 0.001) with 111 cases and 82 expected cases; and the last cluster observed in 2007 consisted of ten villages including: Miwanga, Kimbuga, Ngulagula, Kibiti B, Kinyanya, Kibiti A, Mtawanya, Nyambunda, Mng’aru, Nyambili (RR = 1.86, p = 0.002) with 85 total cases and 58 expected cases. The purely spatial scan for the entire period of 13 years was also identified with seven villages (Mchukwi A, Mchukwi B, Mkupuka, Mangwi, Kibiti A, Nyamwimbe, Mtawanya) with the highest mortality due to malaria rate (Fig. 4).

**Table 3:** Malaria mortality clustering using spatial and space–time analysis in Rufiji and Ifakara HDSS for the study period

Cluster type	Location	Time frame	Circle radius (km)	LLR	Observed cases	Expected cases	RR	P value
Rufiji HDSS								
Spatial analysis								
Mostly likely	Mtawanya, Kinyanya, Pagae, Mchukwi A, Kibiti A, Kibiti B, Nyambili, Nyamwimbe, Mchukwi B, Bungu B	2005	12.09	11.41	112	80.75	2.10	<0.001
Mostly likely	Pagae, Bungu A, Mtawanya, Nyambili, Mchukwi A, Bungu B, Kinyanya, Nyamwimbe, Mlanzi, Kibiti A, Nyambunda	2006	12.16	7.83	111	82.13	1.71	<0.001
Mostly likely	Miwanga, Kimbuga, Ngulagula, Kibiti B, Kinyanya, Kibiti A, Mtawanya, Nyambunda, Mng’aru, Nyambili	2007	18.93	8.44	85	58.29	1.86	0.002
Space time analysis								
Mostly time analysis	Pagae, Bungu A, Mtawanya, Nyambili, Mchukwi A, Bungu B, Kinyanya, Nyamwimbe, Mlanzi, Kibiti A, Nyambunda, Kibiti B	1999–2002	12.43	28.530	536	391.44	1.46	<0.001
Ifakara HDSS (Kilombero)								
Spatial analysis								
Mostly likely	Lukolongo, Mchombe	2008	4.41	4.562	35	21.3	1.87	0.046
Space time analysis								
Mostly likely	Ikule, Mkangawalo, Mngeta, Lukolongoni, mchombe	2007–2009	18.81	22.961	181	109.01	1.82	<0.001
Ifakara HDSS(Ulanga)								
Spatial analysis								
Mostly likely	Igota, Lupiro, Kichangani	2010	3.30	5.302	27	14.96	2.30	0.023
Mostly likely	Kichangani	2012	0	4.255	9	3.03	3.44	0.045
Space time analysis								
Mostly likely	Igota, Lupiro, Kichangani, Igumbiro, Idunda	2007–2009	10.13	11.566	125	81.69	1.66	<0.001

Mostly likely :primary cluster with highest likelihood; secondary cluster is the second cluster followed after mostly likely

*LLR* Log likelihood ratio, *RR* relative risk

In Ifakara HDSS covers parts of Kilombero and Ulanga districts. The clustering for Ifakara HDSS was separated for each district (Table 3; Fig. 4). In Kilombero district part, clusters were observed in 2002–2012. Table 3 shows the purely spatial analysis and indicates one significant cluster in 2008 and involved two villages (Lukolongo and Mchombe) with 35 total malaria deaths cases and 21 expected cases (RR = 1.87, p = 0.046). The purely spatial scan for the entire period of 12 years was also identified with three clusters and the numbers of malaria deaths were not significantly different with expected cases (Fig. 4).

In Ulanga district, clusters were observed in each year from 2002 to 2012. Table 3 shows the purely spatial analysis and indicates two significant clusters, the first significant cluster was observed in 2010 and involved three villages (Igota, Lupiro, Kichangani) with 27 total malaria deaths cases and 17 expected cases (RR = 2.30, p = 0.023); the second significant cluster was observed in 2012 and consisted of one village namely Kichangani (RR = 3.44, p = 0.045) with nine cases and three expected cases. The purely spatial scan for the entire period of 12 years was also identified with two clusters: the first with mostly likely was significantly at three villages (Idunda, Kichangani, Igota) with the highest malaria mortality and secondly at one village (Mavimba) (Fig. 4).

### Temporal trend

**Fig. 5:**
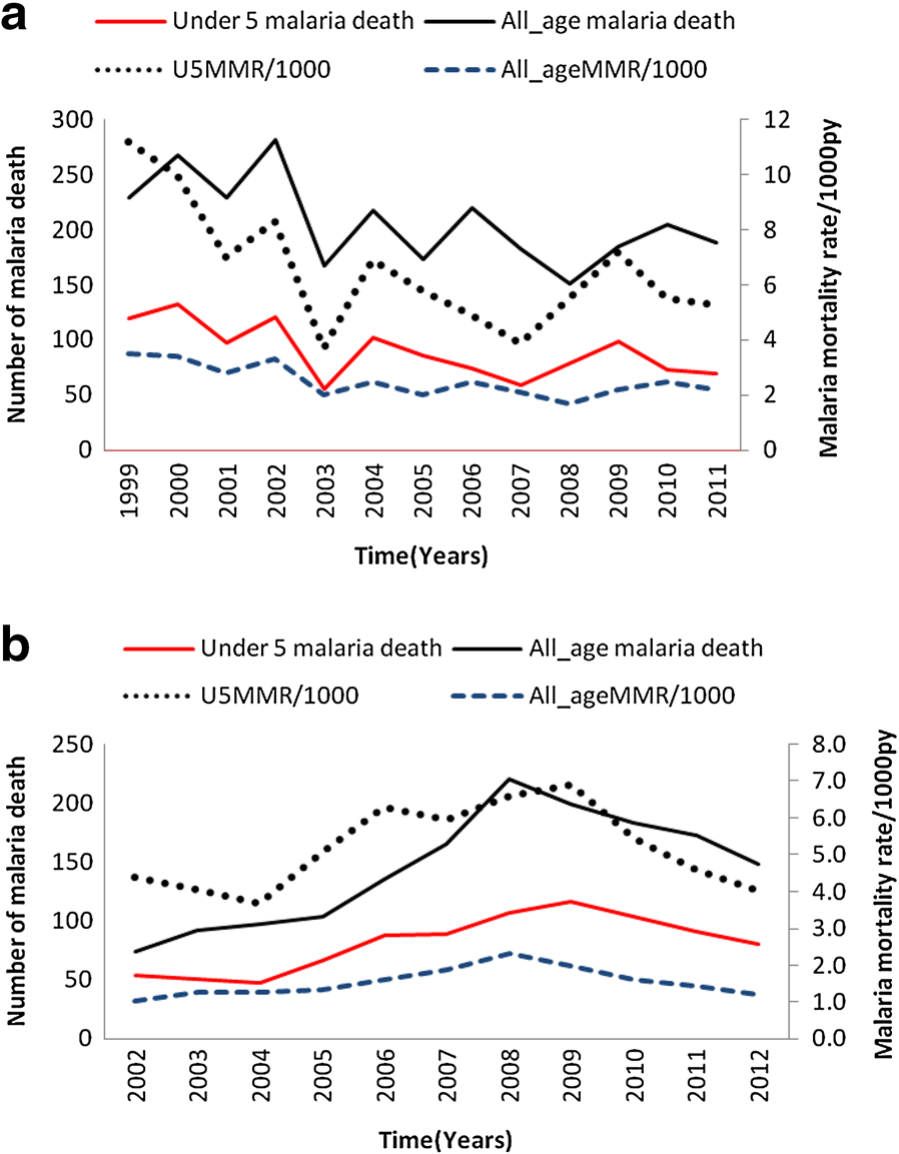
Malaria deaths and estimates of malaria mortality rate in the study area. **a** For Rufiji HDSS and **b** for Ifakara HDSS

Results from purely temporal analysis for high rates showed that 1999–2002 appeared the most likely and significant cluster with high malaria mortality rate (p = 0.001) in Rufiji HDSS. The number of observed malaria deaths in this cluster was 1008 against 778.22 expected cases at a relative risk of 1.47. Temporal trend results show significantly marked decrease in mortality due to malaria in Rufiji HDSS in both under five and all age malaria mortality rate (Fig. 5). Much decrease was from 1999 to 2001 and rose in 2002. Since 2003, there has been a marked and steady decrease in annual mortality due to malaria and malaria deaths. However, there was exceptional high mortality due to malaria in 2004 and 2009.

In Ifakara purely temporal analysis for high rates was run and showed that in Kilombero district, the period of 2007–2009 appeared the most likely and significant cluster with high malaria mortality rate (p = 0.001) was observed. The number of observed malaria deaths in this cluster was 157 and 115.72 expected cases at a relative risk of 1.47. While in Ulanga district the purely temporal analysis for high rates appeared the most likely and significant cluster with high malaria mortality in 2007–2009 with p = 0.001. The number of observed malaria deaths in this cluster was 367 against 269.10 expected cases at a relative risk of 1.59.

From 2008 to 2012 the number of malaria deaths and mortality due to malaria in Ifakara HDSS has been decreasing for both children under-5 years and all ages (Fig. 5). The malaria mortality rate has been decreased from 6.9 per 1000 person-years in 2008 to 4.0 per person-years in 2012 for children under 5 years. Also decrease malaria mortality was observed at all ages from 2.3 (95 % CI: 2.0–2.6) per 1000 person-years to 1.2 (95 % CI: 1.0–1.4) per 1000 person-years in the same period.

### Spatial–temporal clusters

The spatial–temporal analysis using SaTscan™ was run (Table 3) and identified that the significant years were 1999–2002 (p < 0.001) in Rufiji HDSS, which consisted of the twelve villages: Pagae, Bungu A, Mtawanya, Nyambili, Mchukwi A, Bungu B, Kinyanya, Nyamwimbe, (Table 3; Fig. 4) with a relative risk of 1.46. In these areas the observed number of malaria deaths was significantly higher than expected malaria death. One village was consistently observed in significant clusters namely Mtawanya.

The spatial–temporal analysis shows significant years were 2007–2009 (p < 0.001) in Kilombero district, which consisted of the five villages: Ikule, Mkangawalo, Mngeta, Lukolongoni, Mchombe, (Table 3; Fig. 4) with a relative risk of 1.66. Two villages were consistently observed in significant clusters namely Lukolongoni and Mchombe.

In Ifakara HDSS, the spatial–temporal analysis using SaTscan™ was conducted and identified that the significant years were 2007–2009 (p < 0.001) in Ulanga district, which consisted of the five villages: Igota, Lupiro, Kichangani, Igumbiro, Idunda, (Table 3; Fig. 5) with a relative risk of 1.66. One village was consistently observed in significant clusters namely Kichangani.

### Hotspots and coldspots of malaria mortality

The spatial clustering of villages was analyzed into hotspots and coldspots of malaria mortality and significant change over time (see Additional files 1, 2). The Anselin Local Moran’s I showed core hotspots clustering of high malaria mortality for villages next to other villages with high malaria mortality (HMM) and coldspot clusters of low malaria mortality next to other villages with low malaria mortality (LMM) in the study areas. Maps depicting hotspots and high risk areas for significant variables using Getis-Ord statistics were identified (see Additional file 3). These approaches of analysis shows the similar areas with hotspots, defined as areas with statistically significant high malaria mortality consistently located in the significant clusters identified by SaTScan™. This provides for true clustering of malaria mortality indicating heterogeneity and hotspot/coldspot in risk for the study areas.

### Factors associated with malaria mortality

Results of univariate analysis are shown in Table 4 after adjusting village level. Sex, age, ownership of mosquito net, season and altitude were significantly associated with malaria death in Rufiji HDSS while in Ifakara Age, ownership of mosquito net and season was significantly associated with malaria death. Children aged five and above and sex (males) were associated with a significant decrease in the odds of malaria death. Also, ownership of mosquito net and dry season were associated with a decrease in the odds of malaria death in both sites. SES and distance to nearest health facility were not associated with malaria death. The SES was not included in the multivariate modelling.

**Table 4:** Logistic regression model for potential risk factors for malaria death in the study areas

Variable	Rufiji (N = 11462) for 1999–2011				Kilombero/Ulanga (N = 9328) for 2002–2012			
	Univariate		Multivariate		Univariate		Multivariate	
	OR (95 % CI)	P value	AOR (95 % CI)	P value	OR (95 % CI)	P value	AOR (95 % CI)	P value
Sex								
Male (reference)	1.00	0.03		0.604	1.00	0.410		–
Female	1.09 (1.01–1.18)		1.03 (0.93–1.13)		1.06 (0.92–1.20)		–	
Age								
5 and above (reference)	1.00	<0.001	1.00	<0.001	1.00	<0.001		<0.001
Under 5	2.06 (1.85–2.29)		2.04 (1.82–2.28)		2.40 (2.17–2.66)		2.51 (2.25–2.79)	
Ownership of ITN								
No (reference)	1.00	<0.001		<0.001	1.00	<0.001		<0.001
Yes	0.58 (0.52–0.65)		0.57 (0.51–0.64)		0.74 (0.65–0.84)		0.65 (0.57–0.74)	
Social economic status								
Poorest (reference)	1.00	0.345		–	1.00			–
Poorer	1.07 (0.93–1.23)		–		1.22 (1.06–1.41)	0.010	–	
Poor	0.95 (0.81–1.11)	0.496	–	–	1.08 (0.90–1.30)	0.903	–	–
Less poor	1.03 (0.87–1.23)	0.712	–	–	1.09 (0.96–1.26)	0.170	–	–
Least poor	0.91 (0.79–1.06)	0.214	–	–	1.00 (0.84–1.19)	0.993	–	–
Nearest distance to HF								
Less than 5 km (reference)	1.00	0.157		0.516	1.00	0.433		–
5 and above km	1.14 (0.95–1.38)		1.05 (0.90–1.24)		1.09 (0.90–1.33)		–	
Season								
Wet season (reference)	1.00	0.035		0.056	1.00	0.043		0.280
Dry season	0.91 (0.84–0.99)		0.92 (0.85–1.01)		0.88 (0.78–1.00)		0.93 (0.82–1.06)	
Altitude (continuous)	1.001 (1.000–1.0012)	0.003	1.00 (1.000–1.001)	0.002	1.00 (1.00–1.002)	0.710	–	–

Multivariate logistic regression analysis with an adjustment for within village clustering indicated that age and ownership of mosquito net were significantly associated with malaria death in both sites (Table 4). Altitude was additional variable that indicated significant associated with malaria death in Rufiji HDSS. Children age under 5 years were two times (adjusted OR = 2.04, 95 % CI: 1.82–2.28) more likely to die from malaria compared to those aged five and above age in Rufiji. Furthermore, under five age had more than twofold (adjusted OR = 2.51, 95 % CI: 2.25–2.79) increased odds of dying from malaria in Ifakara HDSS compared with five and above age. There was strong evidence that ownership of mosquito net had protective effect for dying from malaria. Households with mosquito net had 43 % (adjusted OR = 0.57, 95 % CI: 0.51–0.64) and 35 % (adjusted OR = 0.65, 95 % CI: 0.57–0.74) lower odds of dying from malaria as compared to those without mosquito net for Rufiji and Ifakara, respectively.

## Discussion

This study has shown consistent villages with malaria mortality clustering in Rufiji and Ifakara HDSS sites for the study period using SaTscan, Anselin’s Local Moran’s I statistic and Getis-Ord statistic (Gi*). These villages were also identified with clustering of all cause mortality for under five children in the same study areas [49, 50]. The clustering of malaria mortality indicates heterogeneity in risk of study areas. Our analysis adds to the existing literature by providing evidence for targeting malaria interventions at small scale areas; previous studies were predominantly for malaria incidence at continental/country level [3–6].

Our findings also indicate that malaria mortality rates started to decline from 2003 in Rufiji HDSS and 2008 in Ifakara HDSS. Space time clustering was observed in 1999–2002 in Rufiji HDSS and 2007–2009 in Ifakara HDSS. The possible explanation for this decline could be attributed to different malaria interventions programs and treatment policies such as change of malaria treatment policy and implementation of integrated management of childhood illness (IMCI) [39, 51] in Rufiji HDSS. The decline coincided with change of malaria first line treatment drugs. In 2002, there was a change in the implementation of national policy of the first-line drug for the treatment of malaria from chloroquine to sulfadoxine pyremethamine (SP) [39]. The impact of the change of treatment policy from chloroquine to SP is large given the higher treatment efficacy of SP upon its introduction [52] and the high resistance to chloroquine before it was replaced [53]. Also there was exceptional high mortality due to malaria in 2004 and 2009. The possible reason for this exceptional is coinciding with the drug resistance to SP in 2004 for malaria treatment [52]. Likewise, the efficacy of the IMCI interventions has been extensively documented [54, 55]. There was also a modest increase in the coverage of mosquito nets over the years (Fig. 3b). All these factors have contributed to a steady decline in malaria mortality within the Rufiji district. Other factors related to improvement in the health services and access to care could explain the decline [56, 57].

In Ifakara HDSS, malaria mortality declined since 2008 for all ages and 2009 for children less than 5 years of age. The decline coincided with the implementation of the first new anti-malaria treatment artemether–lumefantrine (ALU) [40]. The reasons for this delayed fall in malaria mortality in Ifakara are unclear but further examination is warranted to derive lessons for malaria control program elsewhere.

This study used Aselin Local Moran’s I to identify hotspot/coldspot villages in the study areas. The hotspot villages identified with Aselin Local Moran’s I were consistently located in the significant clusters identified by SaTScan™ software using the Martin Kulldorff method. Our findings are consistent with previous studies that have used GIS to analyze malaria situation at micro level for decision making [14]. This study is one of the few studies that have demonstrated the use of spatial statistic tools for malaria mortality clustering [58] in two neighbouring HDSS sites located in three districts in Tanzania. Although results may not be representative of the whole country composed of more than 150 districts, they offer an insight on space–time clustering of malaria mortality at local scales. In addition, because these are HDSS sites, their populations are investigated more often and several health system interventions including malaria interventions were implemented on a research basis than elsewhere in the country [59, 60].

Clustering of high malaria mortality villages next to high ones (HH) in some villages were observed in the study period despite of the recent decline. Villages like Mangwi and Machipi in Rufiji HDSS are relatively remote areas with high forest cover. These villages had relatively lower levels of malaria control interventions; mosquito net coverage for example was lower than elsewhere in the Rufiji HDSS. It was observed that by the end of 2011, about 44 % of households in Mangwi village had owned at least one mosquito net. The villages identified with hotspot (HH) in both sites were the most significant ones, with high incidence of malaria deaths in households without nets (see additional Fig. 3). Access to treatment is also an important indicator of the decrease in malaria mortality and needs to reach remote communities which have an increased risk of malaria infection [11, 27]. This study has also shown that households at greater distances from health facilities had an increased risk of malaria mortality; although there was no statistical evidence, the estimated odds ratio was substantially higher than 1 in both HDSS site. The villages included in the spatial clusters of malaria mortality in this study correspond with previous studies [49, 50]. These studies identified spatial clusters of all cause mortality for under-five children in the same areas.

The villages found to be significantly associated with malaria mortality were repeatedly detected in both the purely spatial and space–time analysis. This may suggest that such villages could have certain underlying characteristics that predisposed them as being high risk areas to malaria mortality. A logistic regression model was therefore used to assess risk factors that determine a household’s characteristic or individual’s risk of malaria mortality. Findings from this study showed that age and ownership of mosquito net were potential factors for malaria mortality in the two sites. Age remained an important factor within the model, with children less than 5 years of age being exposed to a higher risk [58]. Other studies have shown a clear downward trend of the effect of transmission with age which may be an effect of the cumulative malaria exposure [61, 62]. It has also been reported that high cumulative exposure reduces the risk of infection especially in older age [62]. This observation might be associated with the acquisition of malaria immunity which is believed to increase with age or behavioural change of older children [63]. Children in the younger age group are more likely to sleep under insecticide-treated mosquito nets [64] and have proven to be highly protective against malaria. Therefore, at early stages of life, Mosquito nets are beneficial as they might lead to less malaria death and protect children with low (or no) immunity. With time the children build up the immunity and given that the malaria infection is significantly low, the effect of Mosquito nets on their death risks becomes insignificant, which supports the argument that other factors contributed to malaria drives the death in these children [65].

The study has shown that malaria deaths were statistically significantly associated with altitude in Rufiji HDSS. Our findings are consistent with previous studies which reported much less malaria morbidity and mortality in higher altitudes [66, 67]. The overall mean altitude of the Rufiji HDSS is less than 500 m [29]. The possible explanation may be due to the fact that temperature decreases with increasing altitude. In this regard, malaria incidence is said to decrease because of the relationship between temperature and the Plasmodium parasite [66].

The results from this study are comparable to other studies. The observed decline in malaria deaths in the study areas was similar to the decline for malaria deaths reported for Tanzania in world malaria report 2012. The world malaria report shows exception high malaria deaths in 2004 and 2009 as shown in this study [1]. Clustering analysis in this study is similar to the analyses from other HDSS studies in South Africa, Burkina Faso [44, 68]; this study is somewhat compromised by the relatively small size of the study area. Also the results are comparable to the rest of rural Africa and other countries where risk factors identified for malaria have similar picture to other studies as reported in Kenya and India [11, 63].

## Strengths and limitations

The study utilized datasets from Health and Demographic Surveillance System sites which are continuously registered vital demographic events in a geographical defined area. Although few studies have used VA data for investigating malaria cause specific mortality [10–12], verbal autopsy has a great potential for countries like Tanzania where a numerous number of people die from places other than health facilities. With gaps in data on what is killing people because of incomplete vital registration systems [19], VA data provide evidence-based information for health systems decisions and planners for appropriate malaria interventions. VA provide causes of death information at community level that can inform health systems decisions and performance similar to death certification in high-quality hospitals [69]. VAs also complement the health management information system which provides data from health facilities especially in SSA where use of health care is low [69].

This study demonstrates the identification of clusters and hotspots using Kulldorff’s spatial scan statistic, Anselin’s Local Moran’s I statistic and Getis-Ord statistic (Gi*). The study provides strong evidence of their importance for malaria mortality clustering and hotspots. The identification of significant clusters and hotspots can help policy makers and planners to focus for targeted malaria control strategy for elimination of malaria deaths. On the other hand, findings from Health and Demographic Surveillance Systems data provides information to policy makers and program manager which can be translated into policy and practice.

This study has some limitations that need to be considered in interpreting the findings. First, presence of at least one bed net was considered as a proxy for use of bed nets in the house, as information about exact bed nets use was not collected during the study. Secondly, SaTScan™ has limitations that have implication on results interpretation. The circular window imposed on either purely spatial scan statistic or cylindrical window for space–time statistic, usually takes various villages with high malaria mortality rates. If it happens that a village with a low malaria mortality rate is surrounded or is very close to villages with high mortality, the software will then enter this village into the high-mortality cluster villages. However, this limitation does not disqualify SaTScan™ software from its importance in producing summarized information about conversional epidemiological methods of presenting results. Thirdly, there is a potential risk of misclassification of cause of death where the sensitivity and specificity of the VA technique is relatively low. This misclassification may lead to underestimation or overestimation for malaria death. Fourthly, other possible factors associated with malaria deaths (e.g. anti-malaria availability) that was not available in HDSS database was not included in the study, but are found to be more effective against reducing malaria related mortality.

## Conclusions

This study used both SaTScan™ and GIS software which are efficient in processing large epidemiological data at micro level. The study established the role of GIS in disease control as it provided rapid and understandable results which are required for decision making. The distribution and level of malaria deaths presented in this study reveal significant spatial variation in malaria death risks, which previous mapping studies failed to convey.

The findings have important operational relevance to the implementation of the current malaria control strategies in the study area. Targeting malaria control interventions and treatment to hotspots or high risk clusters can help to improve the reduction of malaria death at household and country level. This study has identified several major trends in malaria deaths in Ifakara HDSS over the past period, warranting further investigation.

The study recommends priority control in hotspot villages and high risk areas reported, including consistent significant clustering villages in Rufiji and Ifakara HDSS to address grave malaria situation in the three districts in a cost-effective manner. Reduction in mortality due to malaria calls for more attention to be given to factors that affect malaria deaths the most, such as ownership of mosquito nets and age. Ownership and use of mosquito nets should be a continuous strategy in the study areas, particularly in the high risk areas for better malaria control and prevention of transmission.

## Additional files

10.1186/s12936-015-0905-y Spatio-temporal patterns of malaria mortality rate hotspots and outliers in Rufiji HDSS by Village, 1999–2011.
10.1186/s12936-015-0905-y Spatio-temporal patterns of malaria mortality rate hotspots and outliers in Ifakara HDSS by Village, 2002–2012.
10.1186/s12936-015-0905-y Spatial patterns and intensity of malaria mortality relative to main explanatory variables. The variables displayed are Malaria mortality: A1 for less than five ages, A2 for households without ownership of Mosquito nets at death and A3 for altitude in Rufiji HDSS. B1 for less than five ages and B2 for households without ownership of Mosquito nets at death in Ifakara HDSS.
