# Supplementary figures and images for: Spatial and space–time clustering of mortality due to malaria in rural Tanzania: evidence from Ifakara and Rufiji Health and Demographic Surveillance System sites
Source: Malar J. 2015 Sep 26;14:369. doi: 10.1186/s12936-015-0905-y (PMC4583746; doi:10.1186/s12936-015-0905-y)

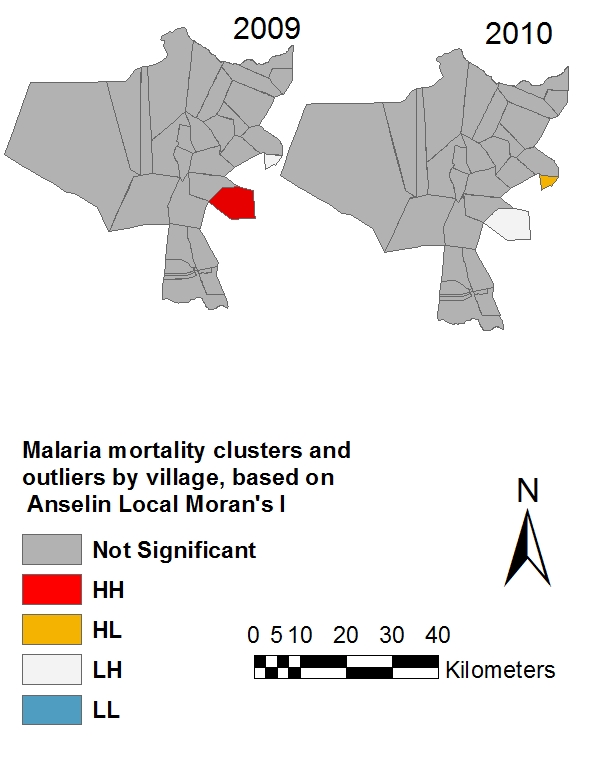

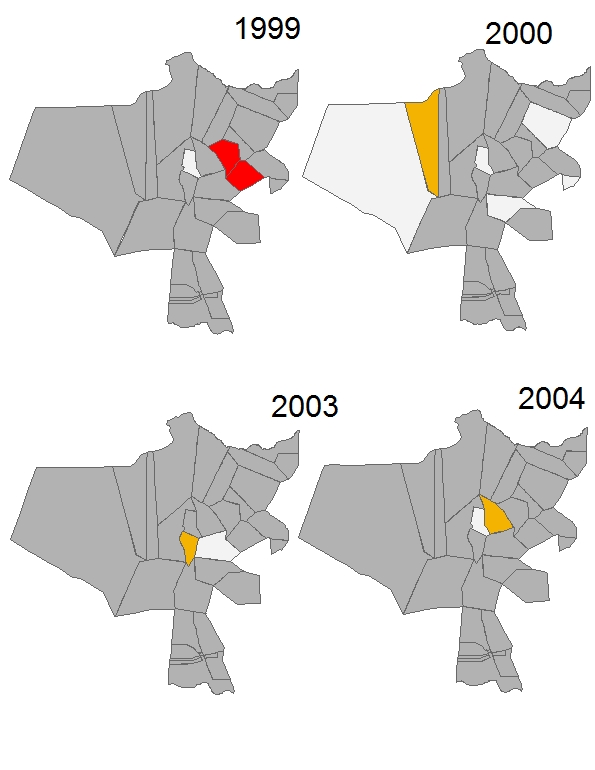

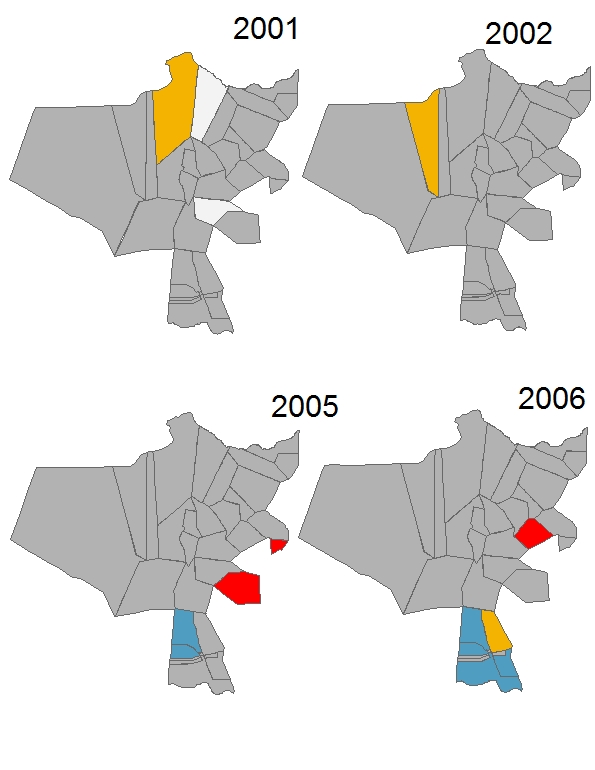

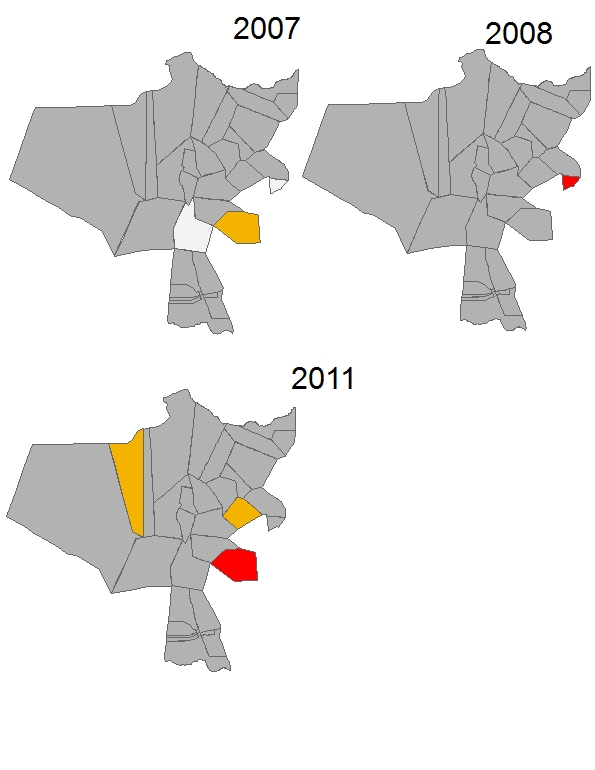

Supplement: Supplementary file 1 — 10.1186/s12936-015-0905-y Spatio-temporal patterns of malaria mortality rate hotspots and outliers in Rufiji HDSS by Village, 1999–2011. [file 12936_2015_905_MOESM1_ESM.doc]

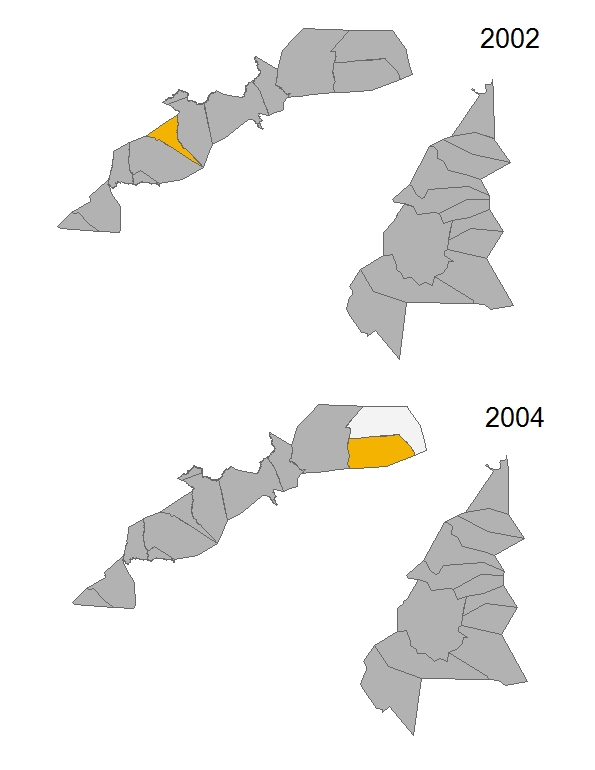

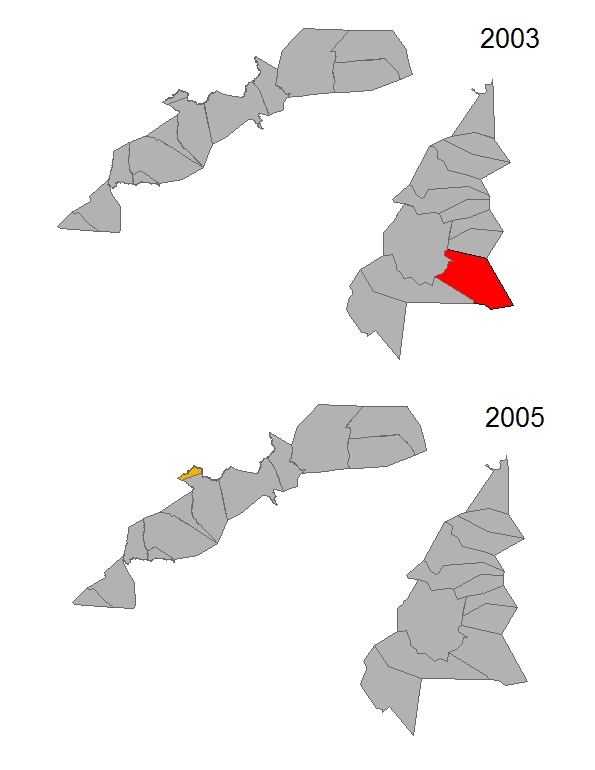


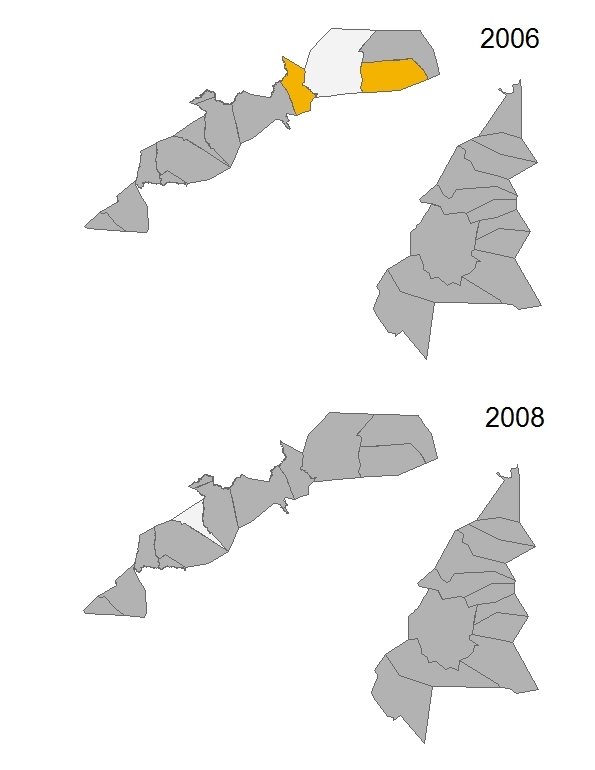

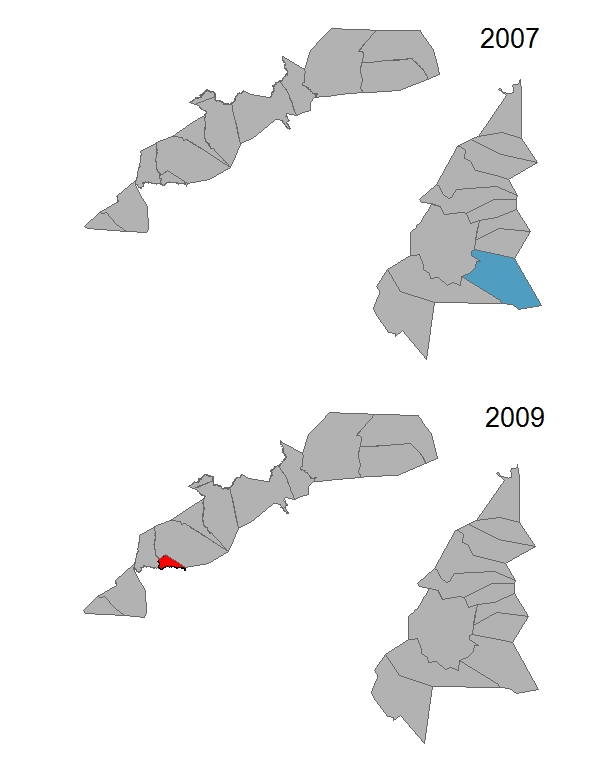

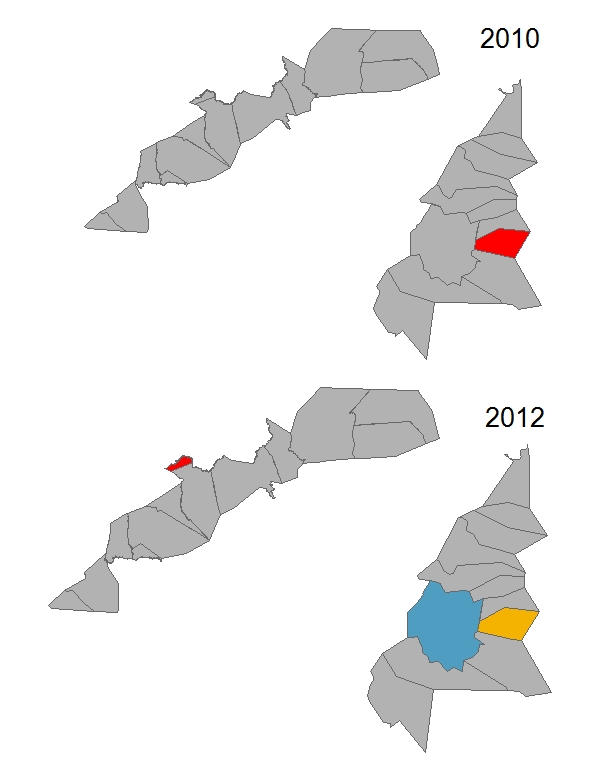

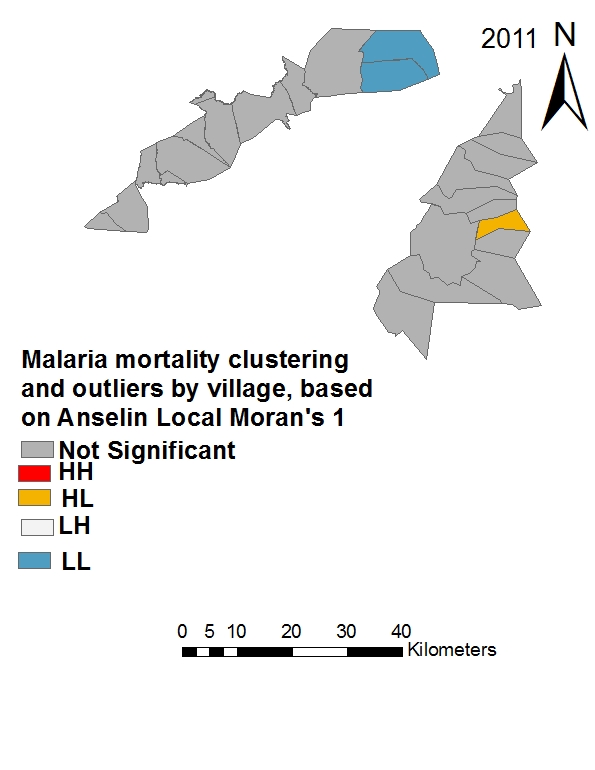

Supplement: Supplementary file 2 — 10.1186/s12936-015-0905-y Spatio-temporal patterns of malaria mortality rate hotspots and outliers in Ifakara HDSS by Village, 2002–2012. [file 12936_2015_905_MOESM2_ESM.doc]

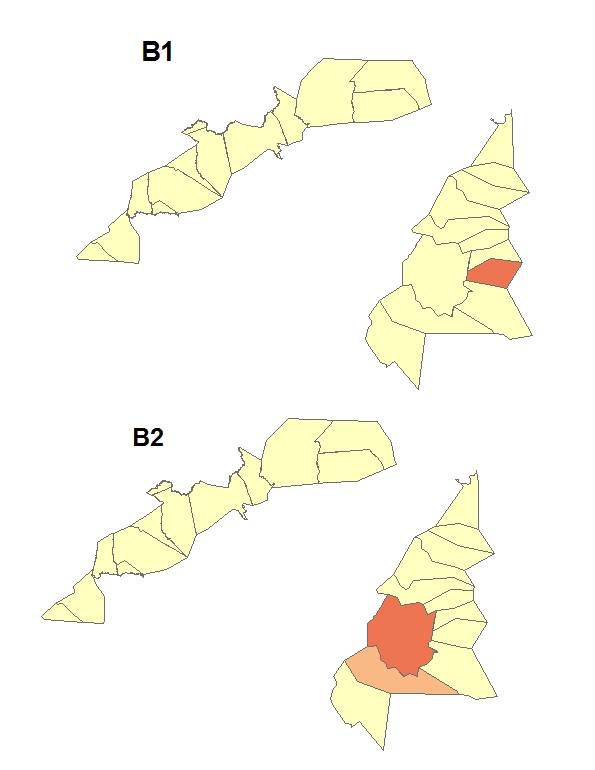

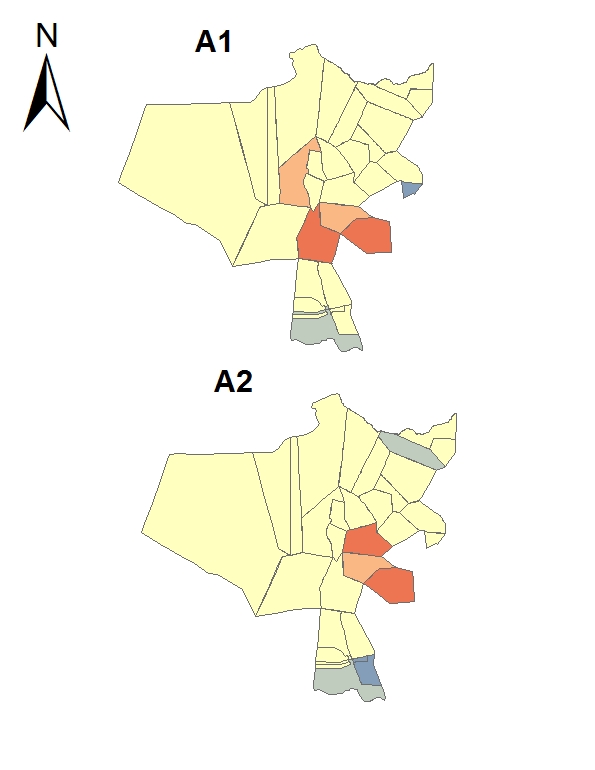

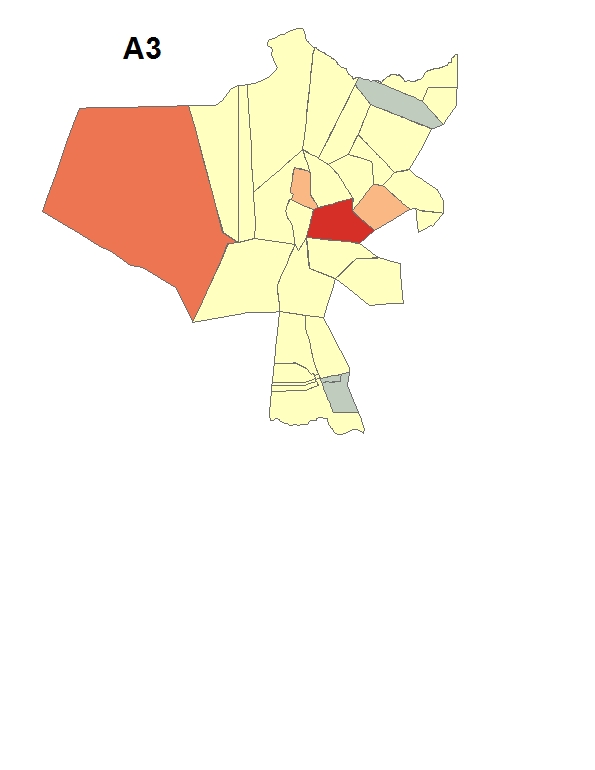

Supplement: Supplementary file 3 — 10.1186/s12936-015-0905-y Spatial patterns and intensity of malaria mortality relative to main explanatory variables. The variables displayed are Malaria mortality: A1 for less than five ages, A2 for households without ownership of Mosquito nets at death and A3 for altitude in Rufiji HDSS. B1 for less than five ages and B2 for households without ownership of Mosquito nets at death in Ifakara HDSS. [file 12936_2015_905_MOESM3_ESM.doc]
